# Combination of Entner-Doudoroff Pathway with MEP Increases Isoprene Production in Engineered *Escherichia coli*

**DOI:** 10.1371/journal.pone.0083290

**Published:** 2013-12-20

**Authors:** Huaiwei Liu, Yuanzhang Sun, Kristine Rose M. Ramos, Grace M. Nisola, Kris Niño G. Valdehuesa, Won–Keun Lee, Si Jae Park, Wook-Jin Chung

**Affiliations:** 1 Energy and Environment Fusion Technology Center, Department of Energy and Biotechnology, Myongji University, Cheoin-gu, Yongin-si, Gyeonggi-do, Republic of Korea; 2 Division of Bioscience and Bioinformatics, Myongji University, Cheoin-gu, Yongin-si, Gyeonggi-do, Republic of Korea; Infectious Disease Research Institute, United States of America

## Abstract

Embden-Meyerhof pathway (EMP) in tandem with 2-*C*-methyl-D-erythritol 4-phosphate pathway (MEP) is commonly used for isoprenoid biosynthesis in *E. coli*. However, this combination has limitations as EMP generates an imbalanced distribution of pyruvate and glyceraldehyde-3-phosphate (G3P). Herein, four glycolytic pathways—EMP, Entner-Doudoroff Pathway (EDP), Pentose Phosphate Pathway (PPP) and Dahms pathway were tested as MEP feeding modules for isoprene production. Results revealed the highest isoprene production from EDP containing modules, wherein pyruvate and G3P were generated simultaneously; isoprene titer and yield were more than three and six times higher than those of the EMP module, respectively. Additionally, the PPP module that generates G3P prior to pyruvate was significantly more effective than the Dahms pathway, in which pyruvate production precedes G3P. In terms of precursor generation and energy/reducing-equivalent supply, EDP+PPP was found to be the ideal feeding module for MEP. These findings may launch a new direction for the optimization of MEP-dependent isoprenoid biosynthesis pathways.

## Introduction

Isoprenoids, also known as terpenes or terpenoids, belong to a large and highly diverse group of compounds which have been utilized or at least have the potential use for biofuels, biopharmaceuticals, nutraceuticals, flavors, fragrances cosmetics, and agrichemicals production [1]–[5]. Isoprene is one of the simplest members of isoprenoids, a valuable starting material for the synthesis of rubber, elastomers and isoprenoid medicines [6]. Isoprene is naturally produced by many plants but due to its volatile nature (b.p. 34°C), its collection from plants is difficult [7]. Hence, much attention and effort have been directed toward isoprene production through metabolically engineered microorganisms [8], [9].

Two metabolic pathways, the mevalonate pathway (MVA) and 2-*C*-methyl-D-erythritol 4-phosphate pathway (MEP), have been intensively studied and applied in the microbial production of isoprene. The MVA is mainly present in eukaryotes and Archaea, which commences with the co-condensation of acetyl-CoA to form acetoacetyl-CoA. Meanwhile, the MEP which starts with the condensation of pyruvate and glyceraldehyde-3-phosphate (G3P) to form 1-deoxy-D-xylulose-5-phosphate (DXP), is typically found in most bacteria. Although these two pathways begin with different precursors, both culminate with the production of two universal 5-carbon isoprenoid precursors, isopentenyl pyrophosphate (IPP) and dimethylallyl pyrophosphate (DMAPP) [10], [11]. The DMAPP can be converted to isoprene by isoprene synthases (IspS), which are cloned from plants and heterologously expressed in microbial hosts [8], [9].

**Figure 1:**
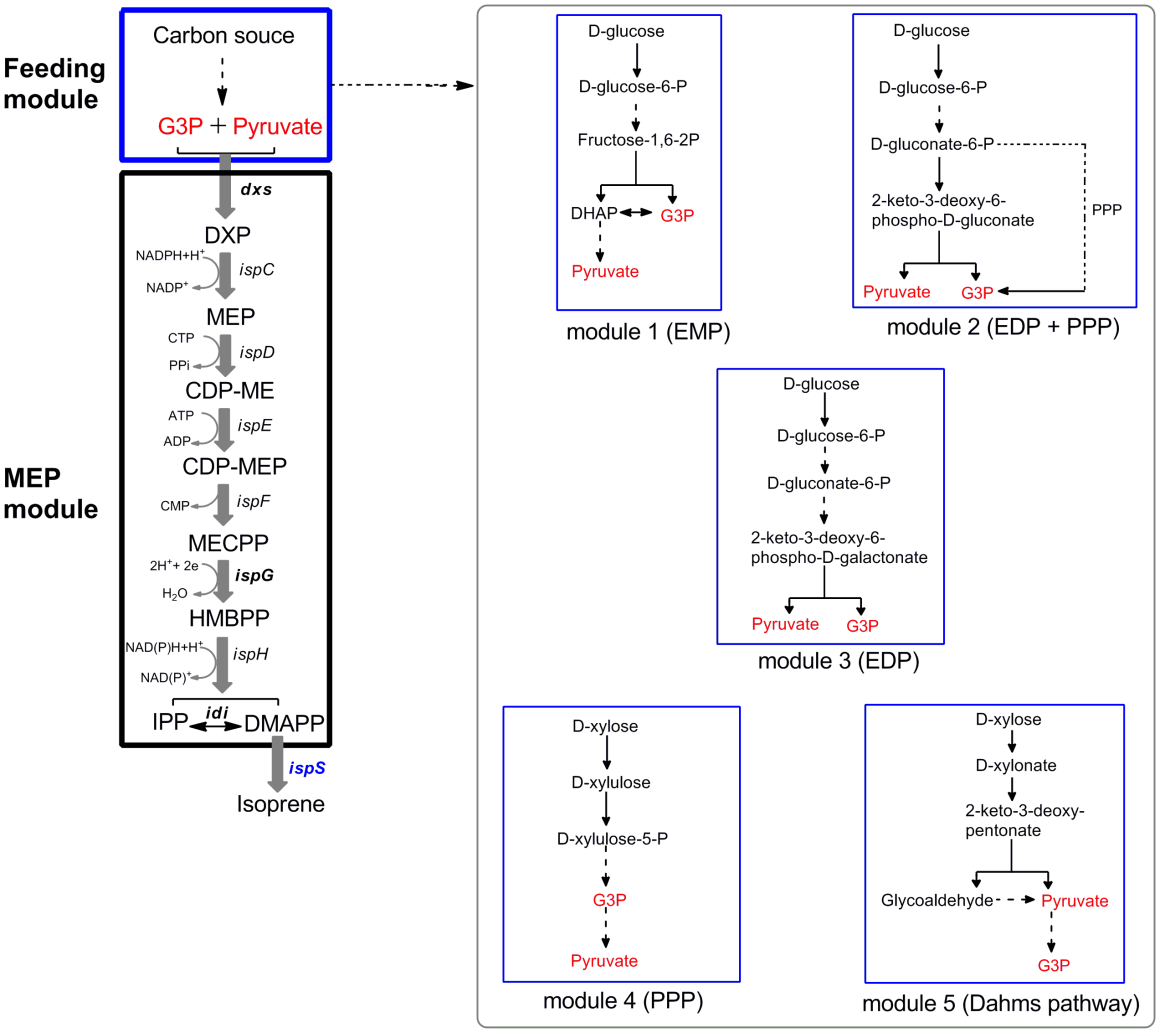
Participation of MEP-dependent isoprene biosynthesis pathway into two modules. Gene symbols and the enzymes they encode (all genes were from *E. coli* except where noted): *dxs*, DXP synthase; *ispC*, DXP reductionisomerase; *ispD*, DXP-ME synthase; *ispE*, CDP-ME kinase; *ispF*, MECPP synthase; *ispG*, HMBPP synthase; *ispH*, HMBPP reductase; *idi*, IPP isomerase; *ispS*, isoprene synthase (*P. alba*). Pathway intermediates: G3P, glyceraldehyde-3-phosphate; DXP, 1-deoxy-D-xylulose 5-phosphate; MEP, 2-*C*-methyl-D-erythritol 4-phosphate; CDP-ME, 4-diphosphocytidyl-2-*C*-methyl-D-erythritol; CDP-MEP, 4-diphosphocytidyl-2-*C*-methyl-D-erythritol 2-phosphate; MECPP, 2-*C*-methyl-D-erythritol 2,4-cyclopyrophosphate; HMBPP, 1-hydroxy-2-methyl-2-(*E*)-butenyl 4-pyrophosphate; IPP, isopentenyl pyrophosphate; DMAPP, dimethylallyl pyrophosphate; DHAP, dihydroxyacetone 3-phosphate.

Although isoprene production from the native MEP in *E. coli* has already been accomplished, the reported titer and yield were low thus require further optimization [12]. In *E. coli*, the whole MEP-dependent isoprenoid biosynthesis pathway can be partitioned into two modules: (1) the feeding module which generates pyruvate and G3P from sugar substrates and (2) the MEP module which produces isoprene as the final product (Figure 1). Previous studies have highly focused on optimizing and balancing the MEP module [1], [8], [12]. On the other hand, only a few studies have focused on modifying the feeding module [13], [14]. Much attention has been given to the Embden-Meyerhof pathway (EMP), the most commonly used feeding module in isoprenoid biosynthesis. These studies sought to balance the distribution between pyruvate and G3P pools by tweaking the EMP. But so far, no effort toward exploiting other feeding modules has been reported.

Thus, this study particularly aims to find a more efficient feeding module for MEP that would improve the isoprene production. Four glycolytic pathways in *E. coli* such as (1) EMP, (2) Entner-Doudoroff pathway (EDP), (3) Pentose Phosphate Pathway (PPP) and (4) Dahms pathway were investigated as MEP feeding modules. These pathways differ in terms of their modes of G3P and pyruvate generation. In EMP and PPP, G3P generation precedes pyruvate whereas Dahms pathway proceeds otherwise, wherein pyruvate is generated prior to G3P. Only EDP simultaneously produces the two MEP precursors in one reaction. Based on these glycolytic pathways, five different feeding modules were constructed by using native or engineered *E. coli* strains; their performances in isoprene production were experimentally compared.

## Materials and Methods

### Strains and plasmids

All strains and plasmids used in this study are listed in Table S1 in File S1. *E. coli* W3110 was purchased from ATCC whereas *E. coli* BW25113 and *E. coli Δgnd::Kan^R^* were obtained from National BioResource Project (NIG, Japan). Construction of *E. coli Δ*xylA was detailed elsewhere previously [15] whereas *E. coli Δpgi* was constructed by deleting *pgi* gene in *E. coli* BW25113. The *pgi* gene disruption cassette was amplified with relevant primers (Table S2 in File S1) using pKD3 as template. *E. coli Δgnd Δpgi* was derived from *E. coli Δgnd::Kan^R^* with *pgi* deletion. Gene disruption and elimination experiments were performed according to the protocols in OPENWETWARE [16]. The plasmid pKD46 was used as the Red recombinase expression vector while pCP20 was used as the resistance gene eliminating plasmid. To express the relevant genes under the control of the T7 promoter, the 69734 λDE3 Lysogenization Kit (Novagen, EMD Millipore, USA) was used to integrate an λDE3 prophage into the *E. coli* host chromosome.

The plasmid *pET28a-xdh* harboring the gene of D-xylose dehydrogenase (Xdh) was constructed according to a previous work [17]. The codon-optimized isoprene synthase gene (*ispS*) of *Populus alba* (GeneBank: AB198180.1) was purchased from Bioneer (South Korea) and was ligated into *pACYCDuet-1* using *Nde*I and *Bgl*II to create *pACYC-ispS*. The DXP synthase gene (*dxs*) was ligated into *pACYC-ispS* using *Bam*HI and *Eco*RI, followed by further ligation of the IPP isomerase gene (*idi*) using *Bgl*II and *Xho*I. This plasmid was denoted as *pACYC-dxs-idi-ispS*. For the construction of *pACYC-dxs-ispG-idi-ispS*, the HMBPP synthase gene (*ispG*) was also ligated into *pACYC-dxs-idi-ispS* using *Sac*I. All primers used for the construction of plasmids were listed in Table S2 in File S1.

### Culture conditions

A 160 mL serum bottle containing 40 mL of semi-defined medium, consisted of M9 salts, 5 g L^−1^ yeast extract, 10 g L^−1^ required carbon source and 1 mM thiamine pyrophosphate (TPP), was utilized for the cultivation of the strains for isoprene production. For strains containing plasmids, relevant antibiotics were also added into the medium. The serum bottle was inoculated with 1 mL of overnight culture and incubated with 150 rpm agitation at 37°C. Isopropyl β-D-1-thiogalactopyranoside (0.5 mM IPTG) was added when the optical density (OD_600_) of the culture reached 0.3 AU. For isoprene accumulation, the serum bottle was sealed with silicone plug after IPTG addition and then transferred into 30°C shaking incubator (150 rpm) for 48 h of cultivation.

### Biomass analysis

Cellular growth was measured in terms of OD_600_. For the calculation of biomass production, a standard curve of dry cell weight was correlated with OD_600_. Samples were collected in 2 mL pre-weighed pre-dried centrifuge tubes and were pelleted at 8,000 *g* for 10 minutes. After discarding the supernatant, the pellets were washed twice with distilled water and dried at 105°C. One OD_600_ unit was equivalent to 0.29 g L^−1^ of dry cell weight.

### Isoprene analysis

Prior to isoprene analysis, the sealed serum bottle was incubated at 60°C for 20 min after the cultivation was finished. Isoprene concentration at the headspace of the serum bottle was analyzed by GC HP6890 equipped with flame ionization detector (FID) and HPFFAP column (50 m×20 µm×34 µm) was used. Nitrogen was used as carrier gas at a linear velocity of 1 mL min^−1^. The column temperature was maintained at 50°C. Identity of the product was confirmed by comparison with a commercial standard (Sigma-Aldrich, USA).

### Carbon source analysis

For carbon source analysis, culture sample was pelleted by centrifugation and the collected aqueous supernatant was analyzed in Waters HPLC equipped with Bio-Rad Aminex HPX-87H Column (300×7.8 mm). The eluent (5 mM H_2_SO_4_) was pumped at a flow rate of 0.4 mL min^−1^. The column temperature was maintained at 55°C and the peaks were detected using a Waters 2414 refractive index detector.

## Results

### Analysis of the four glycolytic pathways as feeding modules

**Figure 2:**
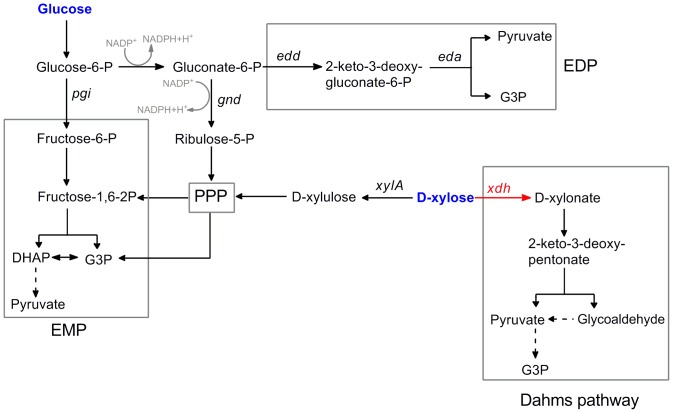
Four glycolytic pathways present in *E. coli*. EMP, Embden-Meyerhof pathway; PPP, pentose phosphate pathway; EDP, Entner-Doudoroff pathway.

The feeding module comprises the carbon flow from a sugar substrate to the two precursors of pyruvate and G3P. In the first step of MEP pathway (Figure 1), one mole of DXP is formed via equimolar condensation of pyruvate and G3P. Thus, limitation in either G3P or pyruvate could sufficiently reduce the DXP production and consequently decrease the isoprene production. In this regard, an ideal feeding module must provide and maintain an equitable balance between pyruvate and G3P pools, as well as supply them at full stream. To meet such criteria, all of the four known glycolytic pathways involved in glucose and/or D-xylose metabolism in *E. coli*, which have been or can be used as MEP feeding modules for isoprene production were investigated (Figure 2).

In native *E. coli*, glucose is mainly metabolized by the EMP pathway, which has been widely used in isoprenoid biosynthesis. However, the main limitation of EMP as MEP feeding module is its imbalanced generation of pyruvate and G3P precursors (Figure 2). Previous studies reveal that further modification of the EMP module to redistribute pyruvate and G3P pools partially increased its efficiency [13], [14], [18]. On the other hand, EDP is another glycolytic pathway responsible for glucose metabolism in *E. coli* [19], [20]. EDP simultaneously produces pyruvate and G3P as it generates the two precursors concurrently with a common cleavage reaction (Figure 2). Thus theoretically, EDP can maintain an equitable distribution between G3P and pyruvate pools, which could be a more efficient MEP feeding module than the EMP. On the other hand, EDP is not active when native *E. coli* strain is grown in the presence of glucose but gluconate addition or blockage of the EMP pathway could activate it [21], [22].

PPP is the sole pathway for D-xylose metabolism in native *E. coli* and is similar to EMP in generating the two precursors; G3P is initially produced followed by pyruvate. Recently, a new D-xylose metabolic pathway, the Dahms pathway, was constructed in *E. coli* K12 family strain [15], [23]. Contrary to PPP, Dahms pathway initially produces pyruvate prior to G3P (Figure 2). Both PPP and Dahms pathway can be used as feeding modules for isoprene production from D-xylose but their efficiencies have not been compared before.

### Construction of five feeding modules

Based on these four glycolytic pathways, five feeding modules were established individually and exclusively in *E. coli*. For feeding module 1, EMP is native in *E. coli* and was hardly affected by other pathways when glucose is used as the sole carbon source. For the construction of feeding module 2, EMP was blocked by disrupting the *pgi* gene in *E. coli* BW25113 to activate EDP. Previous studies indicated that glucose was mainly metabolized through PPP and partially through EDP after *pgi* was disrupted [22], [24]. For the construction of feeding module 3, both *pgi* and *gnd* genes were disrupted to block EMP and PPP, respectively, leaving out EDP as the only available pathway for glucose glycolysis (Figure 2). For feeding module 4, PPP is the only active glycolytic pathway in native *E. coli* when D-xylose is used as the sole carbon source. For the designed feeding module 5, the *xylA* was disrupted to block the PPP for D-xylose metabolism. This was followed by the introduction of a D-xylose dehydrogenase (Xdh) encoding gene from *Caulobacter crescentus* into *xylA*-disrupted strain for the conversion of D-xylose to D-xylonate (Figure 2). As a final step for feeding module 5, an intact Dahms pathway was constructed by combining Xdh encoding gene with *E. coli* native D-xylonate catabolic pathway [15], [25].

It is noteworthy to point out that for each of the feeding module established above, its insulation is a relative concept, i.e. the meaning of insulation is founded on the narrow definition of glycolysis—from a sugar substrate to pyruvate. For instance, in modules 2 and 3, EMP blockage can also affect the carbon flux in TCA cycle and other secondary pathways [22], [26], [27], which could result in the redistribution between pyruvate and G3P pools in the cell. However, since the redistribution occurs at the “post-glycolysis” level, this issue was not considered during the construction of the feeding modules.

### Optimization of MEP module for isoprene production

The codon-adapted *ispS* gene was ligated into *pACYC-duet* vector and expressed in *E. coli* BW25113 (DE3) for the isoprene production. As the control, *E. coli* BW25113 (DE3) host without *ispS* gene was also tested. Results showed that after 48 h of cultivation in M9 medium containing yeast extract and glucose, only <4 mg L^−1^ isoprene was produced by *E. coli* BW25113 (DE3)/*pACYC- ispS*, while none was detected in the control strain. To increase the isoprene production to a level at which the five feeding modules could be appropriately compared, the MEP module was first optimized by overexpressing the *E. coli* native DXP synthase gene *dxs* and isopentenyl diphophate isomerase gene *idi*. This was followed by optimization of the culture medium via 1 mM TPP addition and adjustment in the Carbon: Nitrogen ratio (data not shown). These approaches resulted in an isoprene titer of 35.3±2.5 mg L^−1^. A recent study showed that over-expressing the HMBPP synthase gene (*ispG*) can significantly increase isoprenoid production [28]. Therefore, the *E. coli* native *ispG* was further over-expressed along with *dxs* and *idi*. Integration of all the optimization efforts resulted in isoprene titer of 71.4±1.4 mg L^−1^ (Figure S1). This concentration was easily detected in GC (Figure S2) and was found repeatable with slight fluctuations in shake flask experiment (s.d. <3%), hence can be used for feeding module comparison.

### Comparison of the five feeding modules

**Figure 3:**
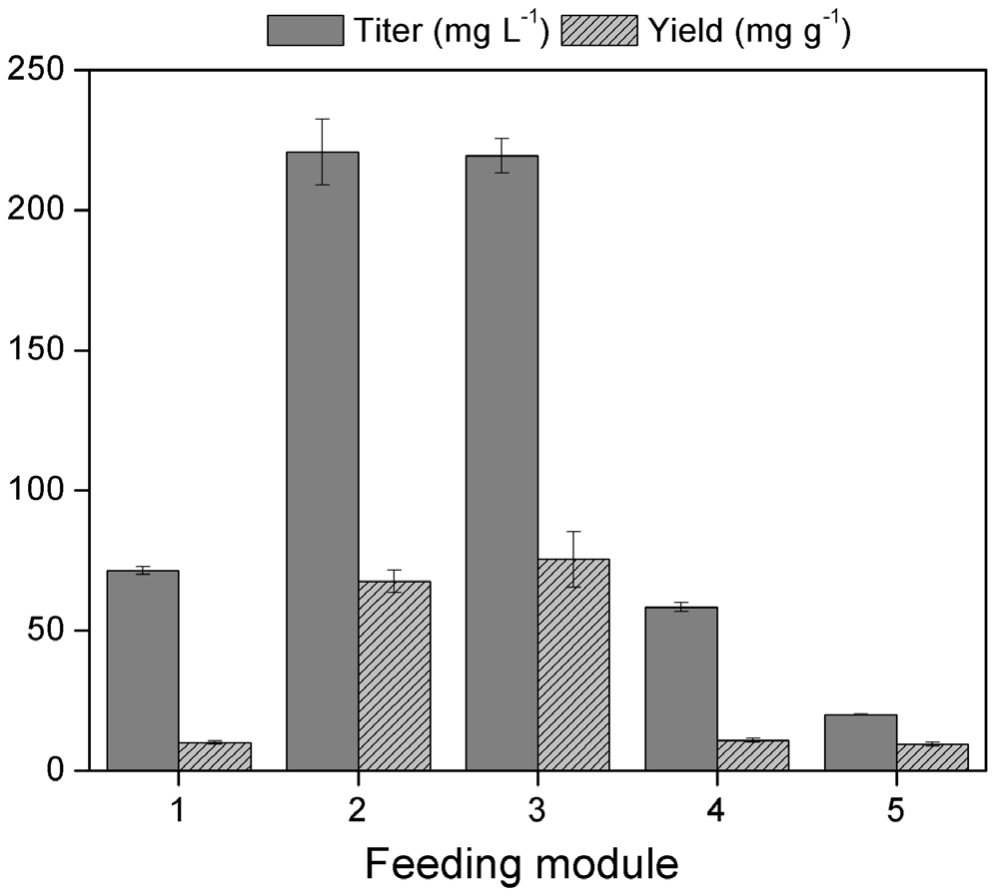
Isoprene titers and yields from different feeding modules. Module 1, EMP of strain FMIS 1; Module 2, EDP+PPP of strain FMIS 2; Module 3, EDP of strain FMIS 3, these three strains used glucose as carbon source. Module 4, PPP of strain FMIS 4; Module 5, Dahms pathway of strain FMIS 5, these two strains used D-xylose as carbon source. All strains are listed in Table S1 in File S1. A 160 mL serum bottle containing 40 mL of semi-defined medium, consisted of M9 salts, 5 g L^−1^ yeast extract, 10 g L^−1^ required carbon source and 1 mM thiamine pyrophosphate (TPP), was used for the cultivation of the strains for isoprene production.

With the optimized MEP module and culture medium, the performance of each of the five feeding modules was tested for isoprene production. Both feeding modules 2 (EDP+PPP) and 3 (EDP) exhibited significant increase in isoprene production (Figure 3). With feeding module 2, the isoprene titer was 3.1 folds higher whereas the yield was 6.8 times better than those of feeding module 1 (EMP). Meanwhile, the titer and yield in feeding module 3 were 3.1 and 7.5 times higher, respectively, than those of module 1. These results attested to the aforementioned hypothesis that the pathways which simultaneously generate pyruvate and G3P could be efficient as MEP feeding modules than the EMP. On the other hand, compared with module 2, no further increase in isoprene production was observed in module 3. This could probably be ascribed to the lower glucose consumption of module 3, which has limited capability of converting more glucose into isoprene (Table 1). Another possibility could be the impairment of the balanced pyruvate and G3P production by EDP through “post-glycolysis” pathways due to the double disruption of *pgi* and *gnd* genes.

**Table 1:** Substrate consumption, isoprene and biomass productions from different feeding modules^a^.

Feeding Module	Strain	Substrate consumption (g L^−1^)	Isoprene production (mg L^−1^)	Biomass production (g L^−1^)
1 – EMP	FMIS 1	7.16±0.62	71.40±1.38	1.20±0.00
2 – EDP + PPP	FMIS 2	3.28±0.12	220.78±11.68	0.81±0.01
3 – EDP	FMIS 3	2.94±0.09	219.42±6.24	0.71±0.06
4 – PPP	FMIS 4	5.40±0.16	58.42±1.68	0.80±0.08
5 – Dahms	FMIS 5	2.11±0.18	20.08±0.13	0.51±0.07

a Module 1, 2 and 3 used 10 g L^−1^ glucose as substrate; module 4 and 5 used 10 g L^−1^ D-xylose as substrate. Data reported were average values of duplicate cultivation runs.

The manner by which G3P is generated prior to pyruvate in module 4 (PPP) resulted in a remarkably higher isoprene production than if pyruvate production precedes G3P as the case in Dahms pathway (feeding module 5). Previous studies indicated that G3P level in the cell is the limit-determining factor for the carbon flux toward MEP hence enhancing the G3P generating flux could increase the isoprenoid production [13], [14]. In Dahms pathway, G3P is produced mainly from pyruvate through gluconeogenesis reactions, which can lower the level of intracellular G3P and consequently result in less isoprene production. These results indicated that for isoprene production from D-xylose, PPP is the preferred feeding module over the Dahms pathway.

## Discussion

Both MVA and MEP have been investigated and exploited for isoprenoid biosynthesis. Stoichiometry and redox balance analysis indicated that MEP is energetically balanced and theoretically more efficient than MVA in converting sugars or glycerol to isoprenoid [11]. On the other hand, recent studies indicated that a heterologous MVA constructed in *E. coli* showed higher efficiency than its native MEP [12], [29], suggesting that more efforts are still required in MEP optimization. Except for common issues of enzyme activities and their internal balance, another obstacle that can hamper MEP efficiency is its necessity to heterogeneously condense two different precursors (pyruvate and G3P), while MVA homogeneously condenses two identical precursors (acetyl-coA). Given that both G3P and pyruvate are individually involved in numerous other pathways, their condensation to DXP rigorously competes with other reactions in the cell. To address the heterogeneous condensation issue, EDP was then applied as the MEP feeding module, as it simultaneously generates G3P and pyruvate. Results showed that this approach remarkably increased the isoprene production. As most studies on MEP-dependent isoprenoid biosynthesis were mainly focused on adjusting the EMP, which remains limited due to its imbalanced generation of the two precursors, the presented strategy in this investigation may launch a new direction for future optimization of MEP-dependent isoprenoid biosynthesis.

**Figure 4:**
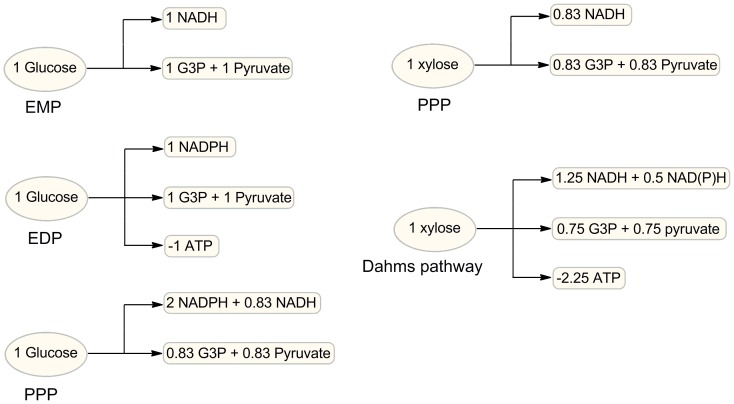
Pyruvate and G3P generation, energy and reducing equivalents production of different glycolytic pathways.

Furthermore, although the four glycolytic pathways have been successfully classified in the perspective of precursor generation with promising results, another important difference among them which could not be neglected is in their energy/reducing equivalent production patterns. As MEP needs both energy (ATP, GTP) and reducing equivalent (NADPH) inputs (Figure 1), the variation in energy/reducing equivalent supply is probably another factor that determines the MEP feeding efficiency of each module (Figure 4). Feeding modules EDP and EMP differ in terms of reducing equivalent generation; the former produces one mole of NADPH whereas the latter produces one mole of NADH. In addition, EMP produces one mole of ATP more than EDP. Albeit lower amounts of precursors are produced when glucose is metabolized through PPP, more reducing equivalents can be generated than those in EMP and EDP. Therefore, based on the trade-off between precursor feeding and energy/reducing equivalent supply, PPP is theoretically considered as a more ideal feeding module than EDP. This might be one of the reasons why feeding module 3 (EDP) has a similar isoprene production with that of module 2 (EDP+PPP).

In addition, regardless of which module (2 or 3) is more efficient, neither has reached its optimum state. Hence, further optimization of these modules is still required. For module 2, aside from PPP, more carbon flux can be directed to EDP by over-expressing *edd* and *eda* genes. This approach may surpass the current trade-off observed between precursor feeding and energy/reducing equivalent supply. For module 3, the whole EDP needs to be enhanced for improved glucose consumption. Overall, the combination of EDP containing feeding modules with MEP has better potentials in isoprenoid production. Further optimization of this combination is expected provide improvement in isoprene biosynthesis.

## Conclusions

The performances of five different MEP feeding modules for isoprene production were analyzed and experimentally compared. Results demonstrated that the feeding modules containing EDP exhibited the highest isoprene production titer and yield, which were significantly higher than those of the widely used EMP. This finding may launch a new direction for further optimization of the MEP-dependent isoprenoid biosynthesis pathways.

## Supporting Information

Figure S1 **Optimization of MEP for improved isoprene production.** A 160 mL serum bottle containing 40 mL of semi-defined medium, consisted of M9 salts, 5 g L^−1^ yeast extract, 10 g L^−1^ Glucose and 1 mM thiamine pyrophosphate (TPP), was utilized for the cultivation of the strains for isoprene production. Data were average values of duplicate cultivation runs. Gene symbol denotes the over-expressed gene in *E. coli* BW25113 (DE3). As the control, *E. coli* BW25113 (DE3) host (without isoprene synthase gene ispS) was also cultivated wherein no isoprene production was detected.
(TIF)

Figure S2 **GC analysis of isoprene production from strain FMIS 1.** A 160 mL serum bottle containing 40 mL of semi-defined medium, consisted of M9 salts, 5 g L^−1^ yeast extract, 10 g L^−1^ Glucose and 1 mM thiamine pyrophosphate (TPP), was utilized for the cultivation of the strain FMIS 1 (*E. coli* BW25113 (DE3)/*pACYC-dxs-ispG-idi-ispS*) for isoprene production.
(TIF)

Table S1 **Plasmids and strains used in this work.**
(DOCX)

Table S2 **Primers used in this study^a^.** ^a^ Straight underline denotes restriction site, waved underline denotes ribosome binding site
(DOCX)

## References

[1] Ajikumar PK, Xiao WH, Tyo KE, Wang Y, Simeon F, et al (2010) Isoprenoid pathway optimization for Taxol precursor overproduction in *Escherichia coli* . Science 330: 70–74. 20929806 10.1126/science.1191652 PMC3034138

[2] Danner H, Boeckler GA, Irmisch S, Yuan JS, Chen F, et al (2011) Four terpene synthases produce major compounds of the gypsy moth feeding-induced volatile blend of *Populus trichocarpa* . Phytochemistry 72: 897–908. 21492885 10.1016/j.phytochem.2011.03.014

[3] Martin VJ, Pitera DJ, Withers ST, Newman JD, Keasling JD (2003) Engineering a mevalonate pathway in *Escherichia coli* for production of terpenoids. Nat Biotechnol 21: 796–802. 12778056 10.1038/nbt833

[4] Ro DK, Paradise EM, Ouellet M, Fisher KJ, Newman KL, et al (2006) Production of the antimalarial drug precursor artemisinic acid in engineered yeast. Nature 440: 940–943. 16612385 10.1038/nature04640

[5] Westfall PJ, Pitera DJ, Lenihan JR, Eng D, Woolard FX, et al (2012) Production of amorphadiene in yeast, and its conversion to dihydroartemisinic acid, precursor to the antimalarial agent artemisinin. Proc Natl Acad Sci USA 109: E111–8. 22247290 10.1073/pnas.1110740109 PMC3271868

[6] Xue J, Ahring BK (2011) Enhancing isoprene production by genetic modification of the 1-deoxy-D-xylulose-5-phosphate pathway in *Bacillus subtilis* . Appl Environ Microbiol 77: 2399–2405. 21296950 10.1128/AEM.02341-10 PMC3067423

[7] Kesselmeier J, Staudt M (1999) Biogenic volatile organic compounds (VOC): an overview on emission, physiology and ecology. J Atmos Chem 33: 23–88.

[8] Lv X, Xu H, Yu H (2013) Significantly enhanced production of isoprene by ordered coexpression of genes *dxs*, *dxr*, and *idi* in *Escherichia coli* . Appl Microbiol Biotechnol 97: 2357–2365. 23143466 10.1007/s00253-012-4485-2

[9] Yang J, Xian M, Su S, Zhao G, Nie Q, et al (2012) Enhancing production of bio-isoprene using hybrid MVA pathway and isoprene synthase in *E. coli* . PLoS One 7: e33509. 22558074 10.1371/journal.pone.0033509 PMC3338741

[10] Misawa N (2011) Pathway engineering for functional isoprenoids. Curr Opin Biotechnol 22: 627–633. 21310602 10.1016/j.copbio.2011.01.002

[11] Yadav VG, De Mey M, Lim CG, Ajikumar PK, Stephanopoulos G (2012) The future of metabolic engineering and synthetic biology: towards a systematic practice. Metab Eng 14: 233–241. 22629571 10.1016/j.ymben.2012.02.001 PMC3615475

[12] Zurbriggen A, Kirst H, Melis A (2012) Isoprene production via the mevalonic acid pathway in *Escherichia coli* (Bacteria). Bioenerg Res 5: 814–828.

[13] Choi HS, Lee SY, Kim TY, Woo HM (2010) In silico identification of gene amplification targets for improvement of lycopene production. Appl Environ Microbiol 76: 3097–3105. 20348305 10.1128/AEM.00115-10 PMC2869140

[14] Farmer WR, Liao JC (2001) Precursor balancing for metabolic engineering of lycopene production in *Escherichia coli* . Biotechnol Prog 17: 57–61. 11170480 10.1021/bp000137t

[15] Liu H, Ramos KR, Valdehuesa KN, Nisola GM, Lee WK, et al (2013) Biosynthesis of ethylene glycol in *Escherichia coli* . Appl Microbiol Biotechnol 97: 3409–3417. 23233208 10.1007/s00253-012-4618-7

[16] Jiang X, Malley K, Jensen HM (2009) Nanobio: Protocol for gene knockout. <URL: http://openwetware.org/index.php?title=NanoBio: Protocol_for_gene_knockout &oldid = 339609> (Accessed 7 June 2010).

[17] Liu H, Valdehuesa KN, Ramos KR, Nisola GM, Chung WJ (2012) High yield production of D-xylonic acid from D-xylose using engineered *Escherichia coli* . Bioresour Technol 115: 244–248. 21917451 10.1016/j.biortech.2011.08.065

[18] Alper H, Jin YS, Moxley JF, Stephanopoulos G (2005) Identifying gene targets for the metabolic engineering of lycopene biosynthesis in *Escherichia coli* . Metab Eng 7: 155–164. 15885614 10.1016/j.ymben.2004.12.003

[19] Flamholz A, Noor E, Bar-Even A, Liebermeister W, Milo R (2013) Glycolytic strategy as a tradeoff between energy yield and protein cost. Proc Natl Acad Sci USA 110: 10039–10044. 23630264 10.1073/pnas.1215283110 PMC3683749

[20] Peekhaus N, Conway T (1998) What's for dinner?: Entner-Doudoroff metabolism in *Escherichia coli* . J Bacteriol 180: 3495–3502. 9657988 10.1128/jb.180.14.3495-3502.1998 PMC107313

[21] Eisenberg RC, Dobrogosz WJ (1967) Gluconate metabolism in *Escherichia coli* . J Bacteriol 93: 941–949. 5337840 10.1128/jb.93.3.941-949.1967 PMC276539

[22] Fong SS, Nanchen A, Palsson BO, Sauer U (2006) Latent pathway activation and increased pathway capacity enable *Escherichia coli* adaptation to loss of key metabolic enzymes. J Biol Chem 281: 8024–8033. 16319065 10.1074/jbc.M510016200

[23] Frost JW, Niu W (2008) Microbial synthesis of D-1,2,4-butanetriol. Patent Pub. No.: WO 2008/091288 A2.

[24] Fraenkel DG, Levisohn SR (1967) Glucose and gluconate metabolism in an *Escherichia coli* mutant lacking phosphoglucose isomerase. J Bacteriol 93: 1571–1578. 5337843 10.1128/jb.93.5.1571-1578.1967 PMC276651

[25] Stephens C, Christen B, Fuchs T, Sundaram V, Watanabe K, et al (2007) Genetic analysis of a novel pathway for D-xylose metabolism in *Caulobacter crescentus* . J Bacteriol 189: 2181–2185. 17172333 10.1128/JB.01438-06 PMC1855722

[26] Charusanti P, Conrad TM, Knight EM, Venkataraman K, Fong NL, et al (2010) Genetic basis of growth adaptation of *Escherichia coli* after deletion of *pgi*, a major metabolic gene. PLoS Genet 6: e1001186. 21079674 10.1371/journal.pgen.1001186 PMC2973815

[27] Zhao J, Baba T, Mori H, Shimizu K (2004) Global metabolic response of *Escherichia coli* to *gnd* or *zwf* gene-knockout, based on ^13^C-labeling experiments and the measurement of enzyme activities. Appl Microbiol Biotechnol 64: 91–98. 14661115 10.1007/s00253-003-1458-5

[28] Zhou K, Zou R, Stephanopoulos G, Too HP (2012) Metabolite profiling identified methylerythritol cyclodiphosphate efflux as a limiting step in microbial isoprenoid production. PLoS One 7: e47513. 23133596 10.1371/journal.pone.0047513 PMC3487848

[29] Pitera DJ, Paddon CJ, Newman JD, Keasling JD (2007) Balancing a heterologous mevalonate pathway for improved isoprenoid production in *Escherichia coli* . Metab Eng 9: 193–207. 17239639 10.1016/j.ymben.2006.11.002

