# Supplementary figures and images for: Combination of Entner-Doudoroff Pathway with MEP Increases Isoprene Production in Engineered Escherichia coli
Source: PLoS One. 2013 Dec 20;8(12):e83290. doi: 10.1371/journal.pone.0083290 (PMC3869766; doi:10.1371/journal.pone.0083290)

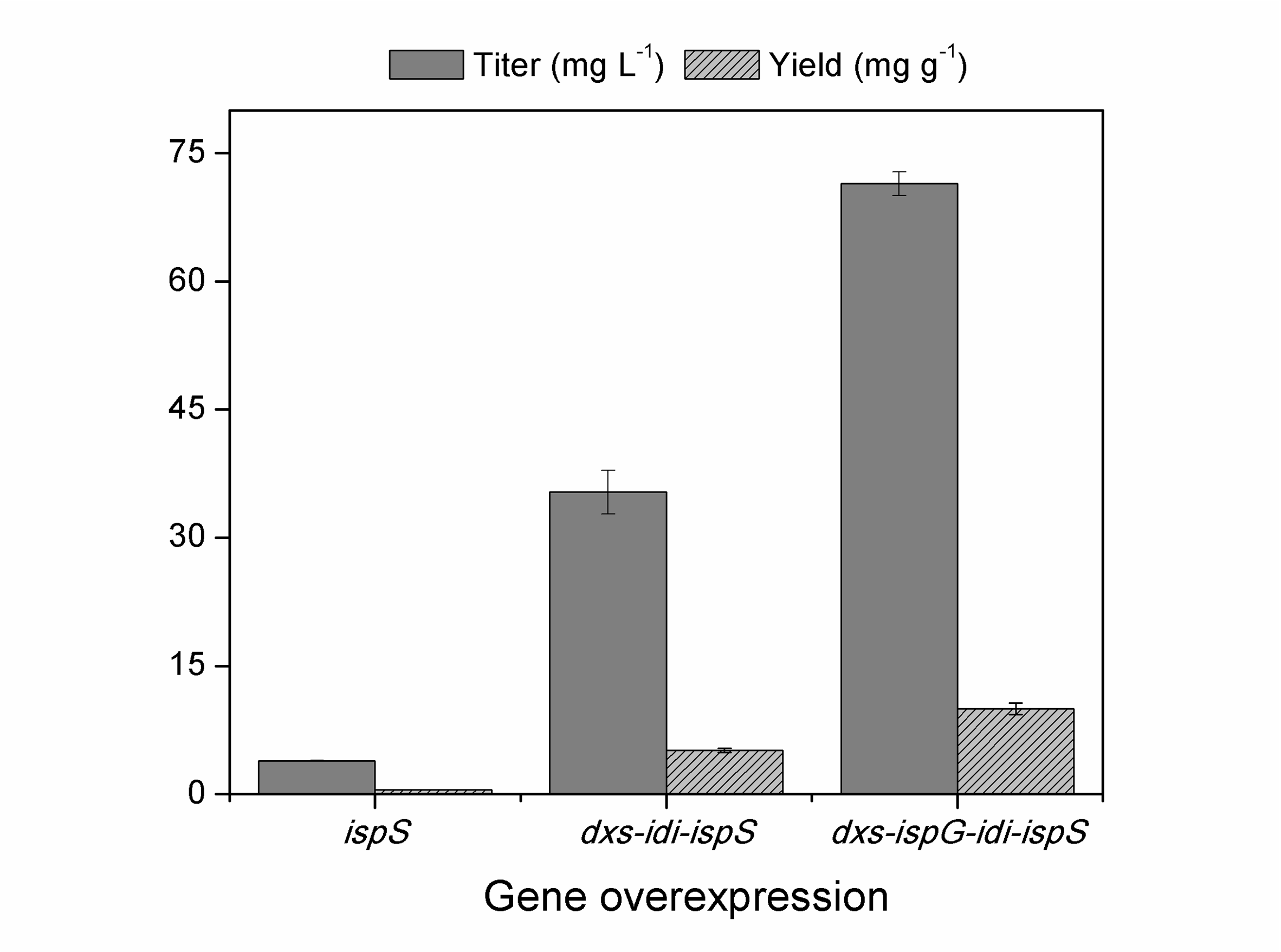

Supplement: Figure S1 — Optimization of MEP for improved isoprene production. A 160 mL serum bottle containing 40 mL of semi-defined medium, consisted of M9 salts, 5 g L−1 yeast extract, 10 g L−1 Glucose and 1 mM thiamine pyrophosphate (TPP), was utilized for the cultivation of the strains for isoprene production. Data were average values of duplicate cultivation runs. Gene symbol denotes the over-expressed gene in E. coli BW25113 (DE3). As the control, E. coli BW25113 (DE3) host (without isoprene synthase gene ispS) was also cultivated wherein no isoprene production was detected. (TIF) [file pone.0083290.s001.tif]
