# Supplementary material for: Combination of Entner-Doudoroff Pathway with MEP Increases Isoprene Production in Engineered Escherichia coli
Source: PLoS One. 2013 Dec 20;8(12):e83290. doi: 10.1371/journal.pone.0083290 (PMC3869766; doi:10.1371/journal.pone.0083290)
Supplement: Table S1 — Plasmids and strains used in this work. (DOCX) [file pone.0083290.s003.docx]

**Table S1. Plasmids and strains used in this work**

| Plasmid/Strain | Function/Characteristic | Resource/Reference |
| --- | --- | --- |
| *pET28a-xdh* | D-xylose dehydrogenase expression vector | Liu et al., 2012 |
| *pACYC-ispS* | Isoprene synthase expression vector | This work |
| *pACYC-dxs-idi-ispS* | DXP synthase, IDI isomerase and isoprene syntase expression vector | This work |
| *pACYC-dxs-ispG-idi-ispS* | DXP synthase, HMBPP synthease, IDI isomerase and isoprene syntase expression vector | This work |
| BW25113 | *E. coli* BW25113F^-^, *Δ(araD-araB)567*, *ΔlacZ4787*(::rrnB-3), *λ^-^*, *rph-1*, *Δ(rhaD-rhaB)568*, *hsdR514* | NBRP No. ME9092 |
| W3110 | *E. coli* W3110 F^-^, *λ^-^* IN (rrnD-rrnE)1 | ATCC No. 27325 |
| *∆pgi* | *E. coli* BW25113 *∆pgi* | This work |
| *∆gnd ∆pgi* | *E. coli* BW25113 *∆pgi ∆gnd* | This work |
| *∆xylA* | *E. coli* W3110 *∆xylA* | Liu et al., 2013 |
| EBW 1 | *E. coli* BW25113 (DE3) / *pACYC- ispS* | This work |
| EBW 2 | *E. coli* BW25113 (DE3) / *pACYC-dxs-idi-ispS* | This work |
| FMIS 1 | *E. coli* BW25113 (DE3) / *pACYC-dxs-ispG-idi-ispS* | This work |
| FMIS 2 | *E. coli* BW25113 (DE3) *∆pgi* / *pACYC-dxs-ispG-idi-ispS* | This work |
| FMIS 3 | *E. coli* BW25113 (DE3) *∆pgi* ∆*gnd* / *pACYC-dxs-idi-ispG-ispS* | This work |
| FMIS 4 | *E. coli* W3110 (DE3) / *pACYC-dxs-ispG-idi-ispS* | This work |
| FMIS 5 | *E. coli* W3110 (DE3) *∆xylA*/ *pET28a-xdh; pACYC-dxs-ispG-idi-ispS* | This work |
