# Supplementary material for: Combination of Entner-Doudoroff Pathway with MEP Increases Isoprene Production in Engineered Escherichia coli
Source: PLoS One. 2013 Dec 20;8(12):e83290. doi: 10.1371/journal.pone.0083290 (PMC3869766; doi:10.1371/journal.pone.0083290)
Supplement: Table S2 — Primers used in this studya. a Straight underline denotes restriction site, waved underline denotes ribosome binding site (DOCX) [file pone.0083290.s004.docx]

**Table S2. Primers used in this study^a^**

| Name | Sequence (5'－3') | Function |
| --- | --- | --- |
| IspS-F | CGATCCATGGATGAGATGTAGCGTGT | For PCR amplification of *ispS* |
| IspS-R | CGTAGATCT TTAGCGAACAAACGGC |  |
| Dxs-F | CGTCGGATCCATGAGTTTTGATATTGCCA | For PCR amplification of *dxs* |
| Dxs-R | CGGGAATTCTTATGCCAGCCAGGCCTTG |  |
| Idi-F | CGTAGATCT AAGGAGATATAATGCAAACGGAACA | For PCR amplification of *idi* |
| Idi-R | CGGCTCGAGTTATTTAAGCTGGGT |  |
| IspG-F | CACGAGCTCAGGAGATATACCATGCATAACCAGGCTCCAAT | For PCR amplification of *ispG* |
| IspG-R | CGTGAGCTCTTATTTTTCAACCTGCTGAACGT |  |
| Pgi-KF | CTTCTCAGAAGCGATTATTTCCGGTGAGTGGAAAGGTTATCATATGAATATCCTCCTTAGT | For PCR amplification of *pgi* disruption cassette |
| Pgi-KR | TACCGTTACGGTCAACATACTTACCGTTGGACTCCATATTGTGTAGGCTGGAGCTGCTTCG |  |
| Pgi-F | TACTCCAAAAACCGCATCAC | For confirmation of *pgi* disruption |
| Pgi-R | CGAAGAAGTTAGACAGCAGT |  |

^a^ Straight underline denotes restriction site, waved underline denotes ribosome binding site
